# Psychological wellbeing in informal workers within the gig economy: the roles of social support, work–life balance, workload, and time pressure

**DOI:** 10.3389/fpsyg.2026.1783219

**Published:** 2026-06-03

**Authors:** Timothy Inan Pongsapan, Muhammad Ramaditya, Astri Warih Anjarwi

**Affiliations:** 1 Faculty of Administrative Sciences, Universitas Indonesia, Depok, West Java, Indonesia; 2 Faculty of Administrative Science, Universitas Brawijaya, Malang, Indonesia

**Keywords:** Gojek drivers, psychological wellbeing, social support, time pressure, work-life balance, workload

## Abstract

This study examines the effect of social support, work-life balance, and workload on psychological wellbeing with time pressure as a mediating variable among Gojek drivers in the Special Capital Region of Jakarta. The research is motivated by the increasing reliance on ride-hailing services within the urban transport system and the growing vulnerability of gig workers’ wellbeing under platform-based work arrangements. Data were collected through an online questionnaire distributed to 267 Gojek drivers who met the predetermined sampling criteria and analyzed using Partial Least Squares Structural Equation Modeling (PLS-SEM) with SmartPLS. The results indicate that social support and work-life balance have a positive and significant effect on psychological wellbeing, while workload has a negative effect. Time pressure is found to significantly mediate the relationship between workload and psychological wellbeing, highlighting its role as a key mechanism through which job demands affect drivers’ mental conditions. These findings underscore the importance of managing job demands and strengthening supportive resources to enhance the psychological wellbeing of Gojek drivers in the context of platform-based work.

## Introduction

Psychological wellbeing has become a central issue in contemporary work research because of its strong association with productivity, engagement, and sustainable performance. Modern organizations no longer treat mental health as a peripheral concern; instead, it is increasingly viewed as a core component of strategic human resource management. This shift reflects the recognition that employees who experience meaning, autonomy, and positive emotions are more resilient and capable of long-term contribution (Guest, 2017; Hannah et al., 2020). Psychological wellbeing therefore goes beyond the absence of psychological distress. It encompasses happiness, life satisfaction, positive functioning, and the pursuit of purpose and personal growth (Diener et al., 2010; Ryff, 1995; Robertson and Flint-Taylor, 2009).

However, empirical evidence shows that many workers still experience declining wellbeing. Deloitte (2023) reports that significant proportions of employees globally feel fatigued, stressed, and emotionally overwhelmed, while leaders often misjudge the severity of these conditions. Similarly, the Workplace wellbeing 360 Report (Intellect, 2025) reveals that Indonesia scores below the global average, particularly in digital and platform-based sectors. These findings indicate that organizational systems and working conditions have not evolved at the same pace as economic and technological change.

The ride-hailing industry illustrates this tension clearly. Rapid technological growth, urbanization, and rising demand for flexible transportation have made ride-hailing platforms essential to urban mobility. Yet platform-based workers frequently operate without the institutional protections associated with traditional employment. Their work is characterized by fluctuating income, algorithmic monitoring, performance ratings, and pressure to remain continuously available. Such dynamics create psychological uncertainty and increase the risk of stress, burnout, and emotional exhaustion (Berger et al., 2019; Gross et al., 2018). In Indonesia, public demonstrations by ride-hailing drivers highlight ongoing dissatisfaction with income security, benefits, and perceived fairness, underscoring that wellbeing concerns are structural rather than individual.

The Job Demands–Resources (JD-R) model provides a useful theoretical lens for understanding these dynamics. Job demands, such as workload and time pressure, generally require sustained physical and psychological effort and, when prolonged, may contribute to strain and reduced wellbeing. Conversely, job resources, such as social support and work-life balance, buffer stress, facilitate coping, and sustain motivation (Bakker and Demerouti, 2017). At the same time, not all demands are necessarily experienced in a purely detrimental way. In certain work settings, especially those characterized by flexibility, performance-based income, and high autonomy, some demands may also be appraised as challenge-like conditions rather than purely harmful stressors (LePine et al., 2005; Crawford et al., 2010). Evidence from gig-economy settings shows that low income, insufficient support, blurred work–life boundaries, heavy workloads, and persistent time pressure significantly shape workers’ psychological wellbeing (Lee et al., 2023). Yet existing research remains concentrated largely on formal employees, public sector workers, or corporate professionals, leaving platform-based workers comparatively understudied.

Within this environment, four variables appear particularly salient: social support, work-life balance, workload, and time pressure. Social support is associated with emotional stability, coping, and resilience, especially where institutional protections are weak. Work-life balance determines whether flexibility becomes a resource or a new form of pressure. Meanwhile, workload and time pressure represent core job demands that may escalate stress and diminish wellbeing when inadequately managed. Understanding how these elements interact is essential to explaining wellbeing outcomes in gig work.

The originality of this research is highlighted by several key distinctions from existing literature. First, while Park and Searcy (2012) and Saragih et al. (2021) establish that job autonomy is a powerful predictor of mental wellbeing and a facilitator of job crafting, their findings are largely rooted in traditional employment or remote work-from-home settings. This study extends these theories to the gig economy, where “autonomy” is often paradoxical—offering flexibility while being simultaneously constrained by algorithmic surveillance.

Second, although recent scholarship has begun to quantify the mental health risks of gig work, such as Wang et al. (2022), who identified loneliness and financial precarity as primary drivers of low life satisfaction, there remains a need to explore the specific mechanisms of work intensity. By integrating workload and time pressure as distinct variables, this research builds upon the “ambivalent subjective experiences” noted by Wu et al. (2022), who found that food-delivery workers often experience a complex mix of satisfaction and strain.

Third, this study moves beyond individual-level psychological factors to consider the role of collective and platform-based support systems. Radzi et al. (2022) and Rahim et al. (2021) emphasize the need for legal protections and registered associations to strengthen the future of the digital labor workforce, particularly post-COVID-19. This research complements those structural perspectives by empirically testing how social support whether from peers or formal networks serves as a psychological buffer against the negative impacts of time pressure.

Finally, by focusing on the Indonesian context (specifically Jakarta), this study responds to the call by Zhang et al. (2022) to “reimagine” algorithmic management by centering worker wellbeing. It contributes to a more global understanding of gig work by providing empirical evidence from a developing economy where informal labor is the primary driver of the transport and logistics sectors, offering a more nuanced view than studies conducted in Western or strictly regulated markets. By synthesizing these perspectives, this study provides a comprehensive framework that addresses not just the presence of stress, but the vital role of work-life balance and social resources in sustaining the psychological health of the modern digital workforce.

## Theoretical background and hypothesis development

### Job Demand-Resource (JD-R) theory

This study is grounded in the Job Demands–Resources (JD-R) model, which explains how job characteristics shape psychological wellbeing and performance (Bakker and Demerouti, 2007). The model distinguishes between job demands, such as workload and time pressure, that require sustained physical and psychological effort and may deplete personal energy (Bakker and Demerouti, 2007), and job resources, such as social support and work-life balance, that help individuals achieve work goals, buffer strain, and facilitate personal growth (Bakker and Demerouti, 2007).

When job demands chronically exceed available resources, psychological strain tends to increase and wellbeing may deteriorate. However, the effects of job demands are not always uniform across work settings. The challenge–hindrance stressor perspective suggests that some demands may be appraised as challenges when they are perceived as meaningful, manageable, or closely linked to personal achievement, whereas others function primarily as hindrances that obstruct functioning and wellbeing (LePine et al., 2005; Crawford et al., 2010). This nuance is particularly relevant in platform-based work, where high activity levels may simultaneously create strain and signal income opportunities.

The JD-R model operates through two main processes. The health-impairment process describes how excessive job demands trigger fatigue, stress, and burnout, ultimately reducing psychological wellbeing (Hakanen et al., 2006). Conversely, the motivational process shows that adequate job resources foster engagement and motivation while mitigating the negative impact of stressors (Tims et al., 2013). Empirical studies further demonstrate that social support protects against depression and anxiety (Soulsby and Bennett, 2015; Fisher and Cassady, 2019), work-life balance reduces role conflict and enhances wellbeing (Schieman et al., 2009), whereas heavy workload and persistent time pressure consistently predict stress, burnout, and poorer health outcomes (Poutanen et al., 2019; Gevers et al., 2001; Silla and Gamero, 2018).

Accordingly, although workload and time pressure are theoretically positioned as job demands in this study, their effects may not be uniformly negative in all platform-based work contexts. The present study therefore remains anchored in the JD-R framework while also recognizing that demands may be interpreted differently depending on workers’ appraisal, autonomy, and access to coping resources. This broader positioning helps situate the current findings without reducing them to a purely impairment-based logic.

Guided by this framework, the present study conceptualizes workload and time pressure as job demands, while social support and work-life balance function as job resources. Psychological wellbeing is positioned as the key outcome arising from the interplay between demands and resources, with time pressure additionally acting as a mediating mechanism linking workload to wellbeing. Accordingly, the JD-R model provides a coherent foundation for examining how these factors interact to shape the psychological wellbeing of Gojek drivers in Jakarta.

### Psychological wellbeing

Psychological wellbeing refers to a psychological condition in which individuals are able to live meaningfully, experience satisfaction with their lives, and realize their full potential. It encompasses more than the mere presence of positive emotions. Ryff (1995) emphasizes that psychological wellbeing involves the pursuit of long-term life goals that allow individuals to develop their optimal capacities and sustain a sense of purpose. In this view, psychological wellbeing is closely tied to self-realization and personal growth that are aligned with one’s values and aspirations.

Ryan and Deci (2001) propose two fundamental perspectives in understanding wellbeing. The hedonic perspective focuses on pleasure, happiness, and subjective life satisfaction, while the eudaimonic perspective highlights meaning, personal development, and the realization of human potential. These perspectives illustrate that psychological wellbeing is a multidimensional construct that integrates emotional experiences with cognitive evaluations of life quality, rather than being limited to momentary feelings of happiness.

More broadly, psychological wellbeing can be understood as the combination of “feeling good” and “functioning well.” It involves the ability to manage challenges, maintain positive relationships, and perform effectively across different life domains (Huppert, 2009). Accordingly, psychological wellbeing reflects a balance between hedonic components such as pleasure and satisfaction, and eudaimonic elements such as growth, authenticity, and meaning (Ryan and Deci, 2001; Quinn and Earnshaw, 2013). This balance serves as a foundation that enables individuals to remain psychologically healthy, adaptive, and resilient in the face of everyday demands.

### Social support

Social support refers to an individual’s belief about the availability and adequacy of assistance from important others, and it is a key determinant of psychological wellbeing because it provides emotional and practical resources (Lakey and Orehek, 2011; Ibarra-Rovillard and Kuiper, 2011). Research shows that social support helps employees manage stress and enhances their psychological functioning (Fisher and Cassady, 2019). Within the JD-R model, it is classified as a job resource that fulfills basic psychological needs and strengthens resilience, which reduces depression and anxiety and promotes higher wellbeing (Bakker and Demerouti, 2007; Hellfeldt et al., 2019). Individuals with stronger perceived support consistently report better wellbeing than those with limited support (Soulsby and Bennett, 2015).

*H1*: Social support influences psychological wellbeing.

### Work-life balance

Work-life balance refers to a condition in which individuals are able to manage work roles and personal life in a harmonious way so that neither domain interferes with the other (Hakim, 2023; Isa and Indrayati, 2023). It is associated with better psychological wellbeing because individuals who successfully balance both spheres experience less role conflict and greater life satisfaction (De Cieri et al., 2002). Autonomy and supportive organizational policies play an important role, since workers who have flexibility and a healthy work environment are more likely to maintain balance and perform effectively (Moore, 2007; Hilman et al., 2022). Time management mechanisms such as flexible scheduling further help individuals coordinate work, family, and personal responsibilities, which reduces role strain and improves motivation and performance (Lappegård et al., 2017; Reimann and Abendroth, 2023; Mihalca et al., 2021).

*H2*: Work-life balance influences psychological wellbeing.

### Workload

Workload refers to an individual’s perception of job demands related to the amount, speed, and difficulty of tasks, and it includes not only quantitative load but also task complexity and intensity that may create uncertainty in meeting deadlines and performance expectations (Spector and Jex, 1998). Within the Job Demands–Resources model, workload is classified as a job demand that drains energy, limits recovery capacity, and increases psychological strain when demands exceed available resources (Bakker and Demerouti, 2007). High workload also interferes with psychological detachment from work, which contributes to emotional exhaustion and poorer work–life balance (Sonnentag and Bayer, 2005). Empirical evidence shows that excessive workload is associated with higher stress and reduced wellbeing, and it leads to outcomes such as work–family conflict, job stress, and dissatisfaction (Bowling et al., 2015; Sadiq, 2020). In modern work settings, digital communication further amplifies these pressures by extending job demands into personal time (Zinke et al., 2023).

*H3*: Workload influences psychological wellbeing.

### Time pressure

**FIGURE 1:**
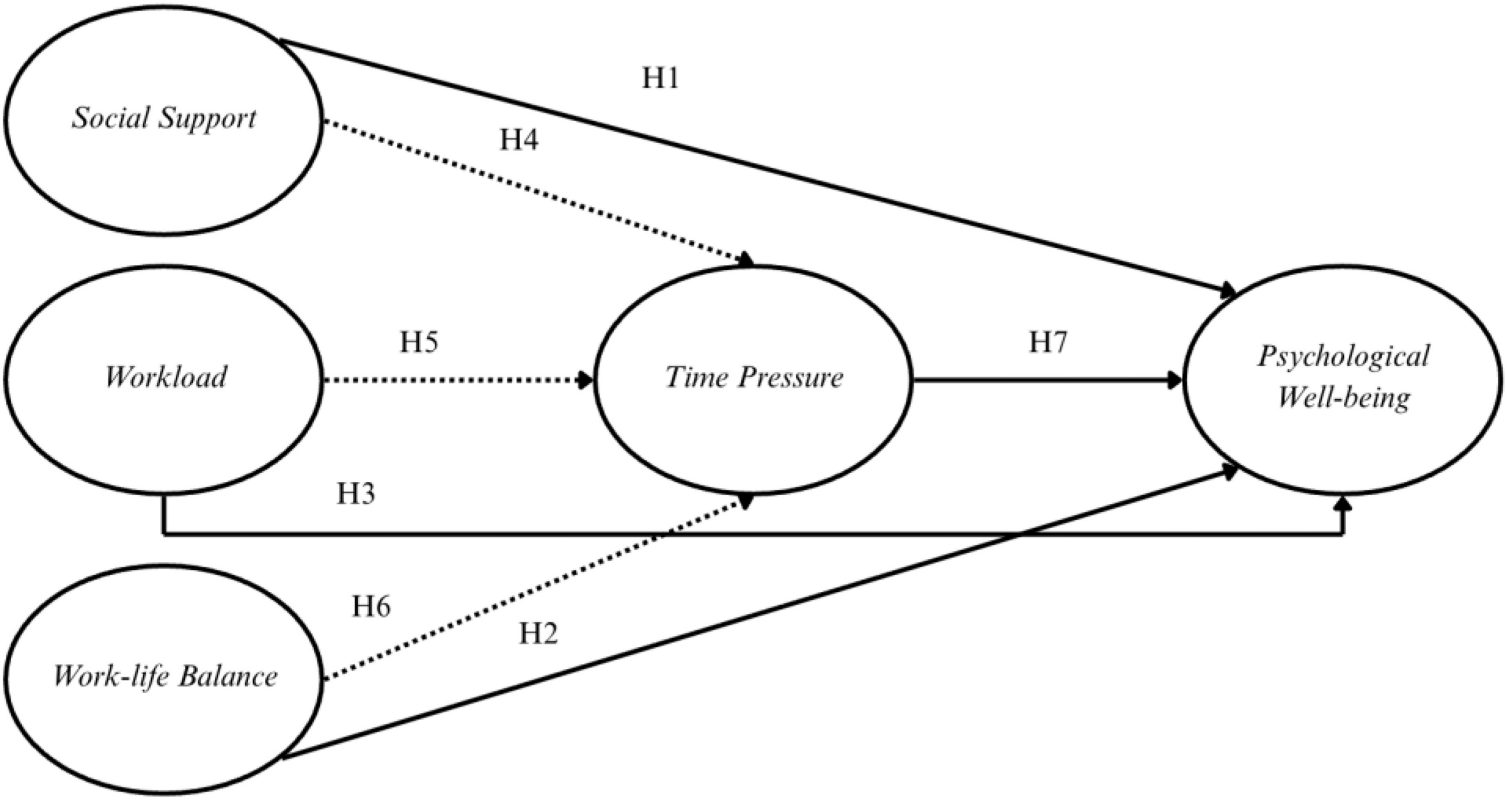
Conceptual model of the study.

Time pressure refers to the condition in which individuals experience limited time to complete tasks, and it often creates tension that may lead to avoidance behavior and negative emotional reactions (Gevers et al., 2001). It is generally considered a work stressor because insufficient time can disrupt goal attainment across work, family, and leisure domains and is often associated with lower emotional wellbeing (Gärling et al., 2016). Within the Job Demands–Resources model, time pressure is classified as a job demand that requires continuous physical and psychological effort and may impose psychological costs when prolonged (Bakker and Demerouti, 2007). However, in certain work environments, especially those characterized by flexibility and strong performance contingencies, time pressure may also be appraised as a manageable challenge rather than solely as a hindrance (Crawford et al., 2010). Figure 1 is the framework of this research.

*H4*: Time Pressure mediates the relationship between social support and psychological wellbeing.

*H5*: Time pressure mediates the relationship between work-life balance and psychological wellbeing.

*H6*: Time pressure mediates the relationship between workload and psychological wellbeing.

*H7:* Time pressure influences psychological wellbeing.

## Methods

The design of this study was quantitative with explanatory and correlational purposes, since the objective was to test theoretical relationships statistically and examine how social support, work-life balance, and workload influence psychological wellbeing, with time pressure acting as a mediating variable. A quantitative approach is appropriate because it allows hypothesis testing and objective interpretation of phenomena through numerical data (Creswell and Creswell, 2023; Neuman, 2019). The study employed a cross-sectional design, meaning that data were collected at one specific time from Gojek drivers operating in Jakarta. Given the highly mobile nature of the driver population and the absence of an identifiable sampling frame, it was not feasible to collect data from all drivers or to apply probability sampling. Therefore, convenience sampling was used to reach respondents who were available and willing to participate at driver bases and operational points. Although this approach was appropriate for accessing a highly mobile informal-worker population, it also limits sample representativeness and introduces the possibility of sampling bias. As a result, the findings should be interpreted with caution in relation to broader generalization beyond the present sample. To determine the minimum sample size, Hair et al. (2020) recommend using at least five respondents per indicator. With 44 indicators included in the model, a minimum of 220 respondents was required. This study is collected from June-September 2025.

In addition, because the study relied on a cross-sectional self-report survey, the findings should be interpreted as associational rather than strictly causal. The use of single-source data also means that common method bias cannot be fully ruled out, although the study attempted to minimize measurement error through questionnaire pre-testing and instrument validation procedures. Jakarta represents a particularly relevant context for examining these mechanisms. As one of the most congested metropolitan areas in Southeast Asia, the city relies heavily on ride-hailing services to bridge gaps in public transportation. Gojek drivers occupy an important socioeconomic role by providing accessible, flexible urban mobility. At the same time, they face prolonged working hours, unpredictable demand patterns, intense competition, and constant evaluation through ratings and algorithmic assignment systems.

Primary data were collected using a structured questionnaire distributed both online through Google Forms and offline through printed questionnaires. The dual method was chosen to ensure inclusiveness, because many drivers were unable to complete online forms during working hours. All items were measured using a five-point Likert scale ranging from 1 (strongly disagree) to 5 (strongly agree). For negatively worded statements, reverse coding was applied so that higher scores consistently reflected more positive perceptions (Babbie, 2020). Before full distribution, a pre-test was conducted with 30 drivers to ensure clarity of wording, alignment between indicators and constructs, and to minimize measurement error (Perneger et al., 2015). Validity testing was then performed using Kaiser-Meyer-Olkin (KMO) and Bartlett’s tests. KMO values greater than 0.50 were considered adequate, while significant Bartlett’s results (*p* < 0.05) indicated that the data were suitable for factor analysis.

This study used Structural Equation Modeling with the Partial Least Squares (PLS-SEM) approach, because it accommodates complex models and does not require strict normality assumptions (Hair et al., 2020). The measurement model assessed convergent validity through factor loadings (≥0.70) and average variance extracted (≥ 0.50), while discriminant validity was evaluated using cross-loadings, the Fornell–Larcker criterion, and HTMT ratios (<0.90). Reliability was confirmed through Cronbach’s alpha (≥0.60) and composite reliability (≥0.70). The structural model then tested path coefficients and *R*^2^-values to determine the strength and direction of relationships among constructs, and mediation effects were examined using bootstrapped specific indirect effects to evaluate whether time pressure transmitted the influence of job resources and job demands on psychological wellbeing (Hair et al., 2020).

### Measurement

Psychological wellbeing was measured using the Flourishing Scale developed by Diener et al. (2010). The scale captures how individuals perceive meaning in life, positive relationships, competence, and optimism about the future, and it is suitable because it offers a comprehensive picture of psychological functioning and personal growth, including feelings of purpose and perceived success in meaningful life domains. In the present study, several items were phrased in a negative direction; therefore, reverse coding was applied prior to analysis so that higher final scores consistently indicated higher psychological wellbeing.

Social support was measured using the revised Multidimensional Perceived Social Support (MPSS) developed by Wu et al. (2025), which is an adaptation of the original instrument by Zimet et al. (1988) that was refined to better fit non-Western cultural contexts. The scale maintains three dimensions, namely family support, friends support, and significant others support, and it evaluates whether individuals feel understood, helped, and emotionally supported by important people in their social environment.

Work-life balance was measured using the Work-Life Balance Scale developed by Hayman (2005). The instrument conceptualizes balance as an interaction between work and personal life, and it includes work interference with personal life, personal life interference with work, and work or personal life enhancement. These dimensions allow researchers to identify whether work disrupts personal functioning, whether personal demands interfere with work activities, and whether both domains can contribute positively to one another.

Workload was measured using the NASA Task Load Index (NASA-TLX), which was developed by Hart and Staveland (1988) and has been widely validated across occupational settings. The instrument evaluates mental demand, physical demand, temporal pressure, perceived performance, effort, and frustration, and therefore reflects both the quantitative and qualitative aspects of workload that individuals experience during their tasks (Hart, 2006).

Time pressure was measured using items adapted from Gärling et al. (2016). The instrument measures the extent to which individuals feel hurried, constrained by time, and unable to complete work or personal activities within available time. It captures perceived pressure across different life domains, and it is therefore suitable for gig workers whose schedules are flexible in structure yet demanding in practice.

## Results

### Outer model evaluation

Based on the Table 1, Cronbach alpha and composite reliability results are presented in Table 2 for Psychological wellbeing (α = 0.880; CR = 0.882), Social Support (α = 0.874; CR = 0.878), Time Pressure (α = 0.865; CR = 0.865), Workload (α = 0.895; CR = 0.896), and Work-life Balance (α = 0.936; CR = 0.937). The outer model evaluation in this study included factor loadings, convergent validity assessment, discriminant validity checks, and reliability testing. The analysis was conducted using partial least squares structural equation modeling (PLS-SEM) with SmartPLS v.4.0 software.

**TABLE 1:** Measurement variables.

Measurement variable	Indicator	Source
Psychological wellbeing	• I feel that my life lacks meaning. • I feel that my social relationships are not supportive or rewarding. • I do not feel engaged or interested in my daily activities. • I rarely play an active role in contributing to others’ happiness and wellbeing. • I feel less capable of carrying out activities that I consider important. • I believe that I am not living my life well. • I feel pessimistic about my future. • I feel that I do not receive enough appreciation from others.	Diener et al. (2010)
Social support	• I feel comfortable talking about work-related problems with my family. • My family tries to help me with my work. • I feel comfortable talking about work-related problems with my friends. • My friends try to help me with my work. • I feel comfortable talking about work-related problems with my significant other. • My significant other tries to help me with my work.	Wu et al. (2025)
Work-life balance	• My job makes it difficult for me to balance my personal activities. • Work demands make my personal needs receive less attention. • My personal activities are often delayed because of my work schedule. • My job reduces the time I have available for my personal life. • Being busy at work makes my personal activities feel less optimal. • My job reduces my flexibility in managing my personal life. • Activities outside of work sometimes affect my ability to focus at work. • My personal responsibilities reduce the energy I have available for work. • My attention to personal matters affects my effectiveness at work. • My personal activities divide my concentration while working. • My personal life gives me positive energy that supports my work. • My job contributes to satisfaction in my personal life. • Activities outside of work create a better mood when I am working. • My work experience helps me develop as a person, not only in work-related matters. • My job provides positive meaning that I also feel in my personal life.	Hayman (2005)
Workload	• My job requires fairly heavy mental activity. • I have to remember a lot of information to complete my tasks. • My job involves physically exhausting activities. • I often feel physically tired after working. • Time pressure makes me rush to complete my work. • I feel satisfied when my work goals are achieved. • Achieving my work goals improves the quality of my work outcomes. • Work demands sometimes make me feel stressed and exhausted. • The effort I put into work requires both mental and physical strength at the same time.	Hart (2006)
Time pressure	• I feel that I do not have enough time to complete my work. • I often feel rushed to finish my work on time. • I feel rushed because the time available to finish my work is not sufficient. • I do not have enough time for activities outside of work. • When I have free time, I often feel restless and pressured to use that time well.	Gärling et al. (2016)

**TABLE 2:** Measurement model.

Construct	Item	Factor Loading	CR	AVE	CA	Source
Social support (SS)	SS_1_1	0.781		0.614		Wu et al. (2025)
	SS_1_2	0.828				
	SS_2_1	0.733				
	SS_2_2	0.778				
	SS_3_1	0.766				
	SS_3_2	0.810				
Work-life balance	WLB_1_1	0.730		0.527		Hayman (2005)
	WLB_1_2	0.721				
	WLB_1_3	0.729				
	WLB_1_4	0.733				
	WLB_1_5	0.795				
	WLB_1_6	0.734				
	WLB_2_1	0.711				
	WLB_2_2	0.708				
	WLB_2_3	0.707				
	WLB_2_4	0.713				
	WLB_3_1	0.717				
	WLB_3_2	0.708				
	WLB_3_3	0.703				
	WLB_3_4	0.758				
	WLB_3_5	0.717				
Workload	WL_1_1	0.725		0.543		Hart (2006)
	WL_1_2	0.776				
	WL_2_1	0.718				
	WL_2_2	0.727				
	WL_3_1	0.718				
	WL_4_1	0.732				
	WL_4_2	0.705				
	WL_5_1	0.751				
	WL_6_1	0.777				
Time pressure	TP_1	0.798		0.651		Gärling et al. (2016)
	TP_2	0.834				
	TP_3	0.834				
	TP_4	0.824				
	TP_5	0.739				
Psychological wellbeing	PWL_1	0.710		0.544		Diener et al. (2010)
	PWL_2	0.792				
	PWL_3	0.709				
	PWL_4	0.707				
	PWL_5	0.763				
	PWL_6	0.733				
	PWL_7	0.781				
	PWL_8	0.701				

Convergent validity was assessed through outer loadings and average variance extracted (AVE). Indicators are considered acceptable when outer loadings are at least 0.70, although values above 0.60 may still be retained in early-stage measurement development if AVE remains adequate, and AVE values should exceed 0.50 (Hair et al., 2020). As reported in Table 2, all indicators in this study showed outer loadings above 0.60, and AVE values ranged from 0.527 to 0.651, which confirms that all constructs meet the minimum criteria for convergent validity (Fornell and Larcker, 1981; Hair et al., 2020).

Discriminant validity was evaluated using three approaches, namely cross loadings, the heterotrait-monotrait ratio (HTMT), and the Fornell–Larcker criterion. Cross loading results (Table 3) indicate that each indicator loads highest on its intended construct compared with other constructs, which supports adequate separation among measures. HTMT values (Table 4) were all below the recommended threshold of 0.90, and the highest HTMT value was observed between Workload and Psychological wellbeing (HTMT = 0.773), while the lowest was between Time Pressure and Social Support (HTMT = 0.503), further confirming discriminant validity (Hair et al., 2020). In addition, the Fornell–Larcker criterion (Table 5) shows that the square root of AVE for each construct exceeded its correlations with other constructs, which provides additional evidence that each construct is empirically distinct (Fornell and Larcker, 1981).

**TABLE 3:** Discriminant validity (cross loadings).

Code	PWL	SS	TP	WL	WLB
PWL_1	0.710	0.427	0.357	0.491	0.489
PWL_2	0.792	0.456	0.490	0.598	0.538
PWL_3	0.709	0.383	0.447	0.476	0.507
PWL_4	0.707	0.397	0.341	0.430	0.420
PWL_5	0.763	0.416	0.451	0.543	0.490
PWL_6	0.733	0.437	0.477	0.535	0.506
PWL_7	0.781	0.402	0.436	0.532	0.471
PWL_8	0.701	0.418	0.425	0.452	0.488
SS_1_1	0.449	0.781	0.423	0.404	0.368
SS_1_2	0.443	0.828	0.293	0.391	0.404
SS_2_1	0.444	0.733	0.264	0.312	0.358
SS_2_2	0.452	0.778	0.456	0.440	0.375
SS_3_1	0.400	0.766	0.262	0.333	0.353
SS_3_2	0.464	0.810	0.354	0.368	0.392
TP_1	0.488	0.358	0.798	0.493	0.496
TP_2	0.433	0.372	0.834	0.427	0.440
TP_3	0.480	0.336	0.834	0.477	0.500
TP_4	0.445	0.400	0.824	0.429	0.458
TP_5	0.494	0.333	0.739	0.557	0.456
WLB_1_1	0.514	0.370	0.448	0.450	0.730
WLB_1_2	0.469	0.402	0.412	0.476	0.721
WLB_1_3	0.450	0.250	0.443	0.384	0.729
WLB_1_4	0.467	0.326	0.420	0.478	0.733
WLB_1_5	0.516	0.368	0.445	0.477	0.795
WLB_1_6	0.465	0.369	0.412	0.406	0.734
WLB_2_1	0.491	0.383	0.520	0.448	0.711
WLB_2_2	0.510	0.376	0.430	0.501	0.708
WLB_2_3	0.480	0.388	0.361	0.402	0.707
WLB_2_4	0.469	0.300	0.400	0.389	0.713
WLB_3_1	0.446	0.290	0.414	0.357	0.717
WLB_3_2	0.479	0.314	0.363	0.381	0.708
WLB_3_3	0.479	0.361	0.380	0.397	0.703
WLB_3_4	0.512	0.385	0.494	0.445	0.758
WLB_3_5	0.472	0.323	0.399	0.460	0.717
WL_1_1	0.486	0.419	0.385	0.725	0.403
WL_1_2	0.552	0.366	0.476	0.776	0.523
WL_2_1	0.524	0.297	0.495	0.718	0.390
WL_2_2	0.462	0.403	0.420	0.727	0.394
WL_3_1	0.508	0.321	0.417	0.718	0.452
WL_4_1	0.470	0.295	0.418	0.732	0.356
WL_4_2	0.478	0.324	0.396	0.705	0.403
WL_5_1	0.521	0.391	0.451	0.751	0.503
WL_6_1	0.569	0.389	0.473	0.777	0.497

**TABLE 4:** Discriminant validity for HTMT.

	PWL	SS	TP	WL	WLB
PWL	0.643	0.503	0.669	0.644	
SS					
TP	0.662				
WL	0.773	0.541			
WLB	0.729	0.528	0.645		

**TABLE 5:** Discriminant validity (Fornell-Larcker criterion).

Construct	PWL	SS	TP	WL	WLB
PWL	0.738	0.783	0.807	0.737	0.726
SS	0.566				
TP	0.583	0.446			
WL	0.691	0.483	0.595		
WLB	0.664	0.479	0.585	0.594	

Overall, the outer model results demonstrate that the measurement model is both valid and reliable. All constructs achieved acceptable internal consistency, and they met the recommended criteria for convergent and discriminant validity, indicating that the indicators appropriately represent the latent variables examined in this study.

### Inner model evaluation and hypothesis testing

**FIGURE 2:**
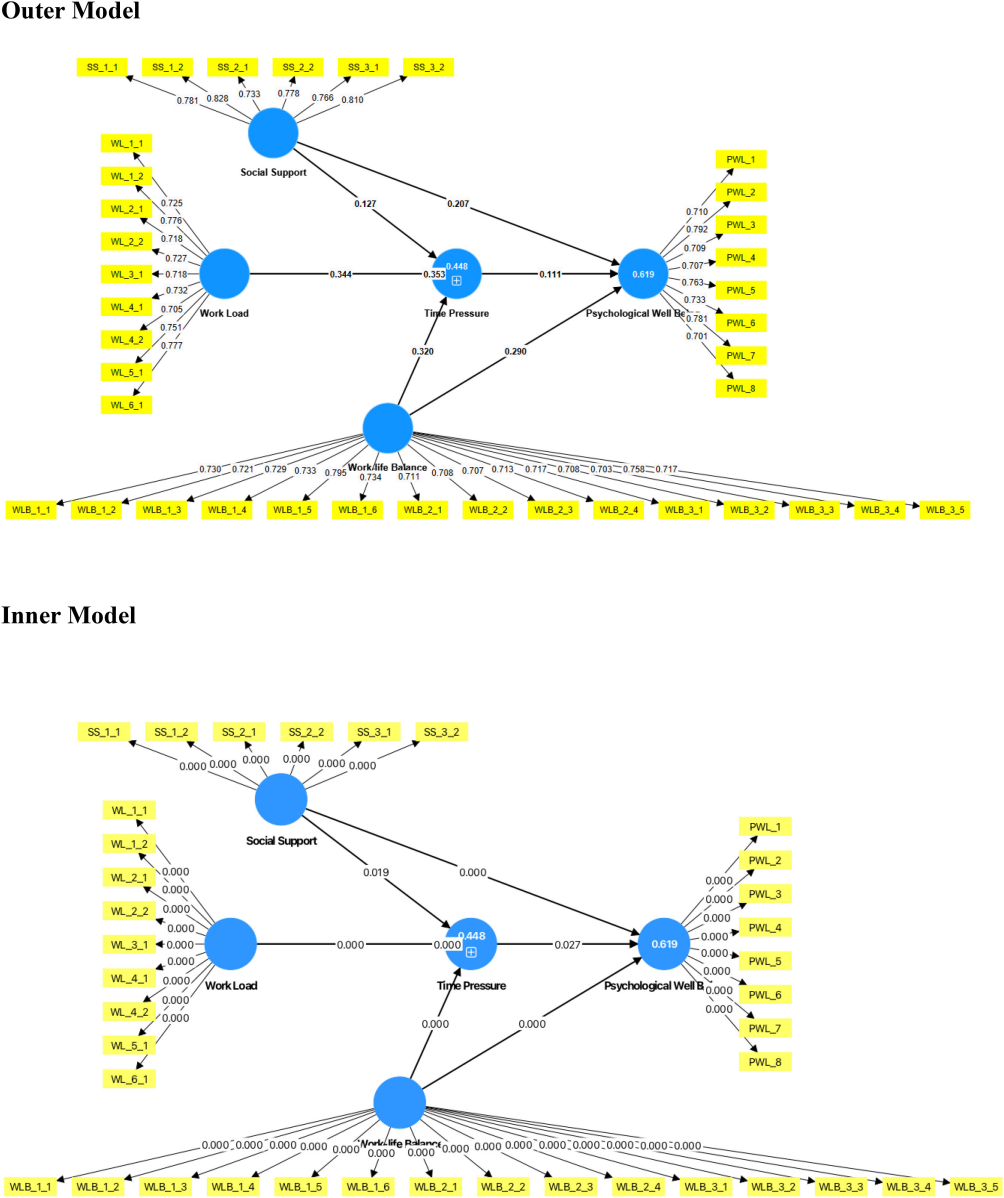
Structural equation model.

After the measurement model was confirmed to be valid and reliable, the structural model was evaluated using PLS-SEM in SmartPLS 4.0 to assess the hypothesized relationships among the constructs. Following Hair et al. (2020), the structural model evaluation in this study focused on the coefficient of determination (R^2^) to assess the explanatory power of the model and on path coefficient estimates obtained through bootstrapping to examine the direction and significance of both direct and indirect effects (Hair and Alamer, 2022). In this model, psychological wellbeing was specified as the endogenous construct, while social support, work-life balance, and workload were modeled as exogenous predictors, with time pressure positioned as a mediating variable. Table 6 presents the coefficient of determination (R^2^) results, Table 7 reports the SEM path coefficient estimates and hypothesis testing results, and Figure 2 illustrates the structural model.

**TABLE 6:** Coefficient of determination (PLS Method).

Relationship	*T*-value	Direct effect (β)	*p*-value
SS → PWL	4.176	0.207	0.000
WLB → PWL	5.076	0.290	0.000
WL → PWL	4.457	0.353	0.000
SS → TP → PWL (mediates)	1.666	0.014	0.096
WLB → TP → PWL (mediates)	2.084	0.036	0.037
WL → TP → PWL (mediates)	1.980	0.038	0.048
TP → PWL	2.219	0.111	0.027

**TABLE 7:** Results of the structural equations model.

Construct	*R* ^2^	*R*^2^ adjusted
Psychological wellbeing	0.619	0.613

As shown in Table 6, the *R*^2^-value for Psychological wellbeing was 0.619, indicating that social support, work-life balance, workload, and time pressure jointly explained 61.9% of the variance in psychological wellbeing among Gojek drivers in Jakarta. This value suggests a substantial level of explanatory power for the proposed model (Hair et al., 2020). Hypothesis testing was conducted by examining standardized path coefficients (β), t-statistics, and *p*-values obtained from the bootstrapping procedure, where relationships were considered statistically significant when *t*-values exceeded 1.96 and *p*-values were below 0.05 (Hair et al., 2020; Sarstedt et al., 2017).

Results provide support for the direct effects in the model. As reported in Table 7, social support had a positive and significant effect on psychological wellbeing (β = 0.207, *t* = 4.176, *p* = 0.000), and work-life balance also showed a positive and significant effect on psychological wellbeing (β = 0.290, *t* = 5.076, *p* = 0.000). Workload was positively and significantly related to psychological wellbeing (β = 0.353, *t* = 4.457, *p* = 0.000), and time pressure similarly demonstrated a positive and significant effect on psychological wellbeing (β = 0.111, *t* = 2.219, *p* = 0.027). These findings indicate that higher levels of job resources were associated with better reported psychological wellbeing in the sample. At the same time, the positive coefficients for workload and time pressure should be interpreted cautiously, as they may reflect the specific context of platform-based work in which some demands are appraised as manageable, meaningful, or income-related rather than purely harmful.

The mediation analysis was assessed using the specific indirect effects generated through bootstrapping. The indirect effect of social support on psychological wellbeing via time pressure was not statistically significant (β = 0.014, *t* = 1.666, *p* = 0.096), indicating that time pressure did not mediate the social support and psychological wellbeing relationship. In contrast, time pressure significantly mediated the relationship between workload and psychological wellbeing (β = 0.038, *t* = 1.980, *p* = 0.048) and also mediated the relationship between work-life balance and psychological wellbeing (β = 0.036, *t* = 2.084, *p* = 0.037), as summarized in Table 7. These indirect effects should be interpreted with caution, because they do not fully reflect the conventional health-impairment pathway assumed in the JD-R model. Rather than indicating that time pressure is inherently beneficial, the results may suggest that, in this context, time urgency is not always experienced as harmful and may partly reflect an adaptive response to platform-based work demands. Figure 2 provides a visual overview of the tested structural relationships and the standardized path estimates.

## Discussion

This study set out to explain why some Gojek drivers in Jakarta are able to maintain psychological wellbeing despite working in conditions that are often fast-paced and demanding. Specifically, it examined how social support, work-life balance, and workload relate to wellbeing, and whether time pressure helps clarify the pathway through which those factors matter (Healy et al., 2017). Taken together, the findings portray driver wellbeing as something shaped by both “what helps” and “what strains,” but not in a simplistic way. Social support and work-life balance consistently appear as stabilizing forces, while workload and time pressure do not automatically translate into poorer wellbeing in this setting. This suggests that in platform-based work, demanding conditions may be experienced as manageable when drivers still feel supported, can regulate their boundaries, and perceive their effort as producing meaningful outcomes.

From a theoretical angle, the results provide a contextual refinement of the Job Demands–Resources (JD-R) model in platform-based informal work. Rather than simply replicating JD-R in a new setting, the study suggests that the meaning of job demands may be more context-sensitive than conventional formulations often assume. In particular, the findings indicate that some demands in gig work may be interpreted not only as strain-producing conditions but also as challenge-like experiences when they are tied to autonomy, productivity, or income opportunity (Bakker and Demerouti, 2007). The positive role of social support and work-life balance aligns with JD-R’s view that resources strengthen psychological functioning by buffering strain and fostering engagement (Bakker and Demerouti, 2007). Prior evidence similarly shows that supportive relationships contribute to wellbeing by reducing vulnerability to distress and reinforcing feelings of belonging, while work-life balance lowers role conflict and supports healthier functioning across life domains (Soulsby and Bennett, 2015; Fisher and Cassady, 2019; Schieman et al., 2009). At the same time, the pattern involving workload and time pressure highlights a nuance often emphasized in the challenge–hindrance perspective: certain demands can coexist with positive outcomes when they are appraised as challenges, remain within a controllable range, and are paired with adequate recovery or coping strategies (LePine et al., 2005; Crawford et al., 2010). In other words, the JD-R “health-impairment” pathway is not inevitable, particularly when drivers interpret high activity as progress, competence, or achievement rather than as chronic overload.

Empirically, these findings both echo and refine what has been reported in earlier wellbeing research. The association between social support and stronger psychological wellbeing is consistent with evidence that perceived support has protective value and is especially important for workers operating under uncertainty (Soulsby and Bennett, 2015; Fisher and Cassady, 2019). This is also in line with studies indicating that limited support can be a risk factor for poorer wellbeing among gig workers (Lee et al., 2023). The positive contribution of work-life balance similarly fits the broader literature showing that boundary management and recovery opportunities help individuals sustain wellbeing under demanding conditions (Schieman et al., 2009; Sonnentag and Fritz, 2007). The more distinctive insight lies in how workload and time pressure may function in this context. Rather than acting purely as stressors, they may sometimes operate as signals of challenge and momentum, particularly when drivers are able to mentally disengage during rest periods and when the work structure still allows some self-regulation (LePine et al., 2005; Sonnentag and Bayer, 2005).

The role of time pressure also adds a useful layer of interpretation. The findings suggest that time pressure is not simply a universal conduit through which all predictors influence wellbeing. Instead, it appears more closely tied to factors that shape daily rhythm and boundary control (Hallward-Driemeier and Gajigo, 2015). This helps explain why work-life balance, and in certain ways workload, can be meaningfully connected to time-pressure dynamics, while social support may influence wellbeing more directly through emotional reassurance and psychological security without necessarily changing the tempo imposed by the platform environment (Bakker and Demerouti, 2007). Put differently, supportive ties can help drivers feel steadier, but they may not materially reduce the structural features of gig work that generate urgency (Halbesleben and Buckley, 2004). This distinction aligns with the view that some pressures are embedded in the work system and cannot be fully “removed” through interpersonal resources alone, even when those resources still improve how individuals cope and function (Tannous-Haddad et al., 2024).

Practically, the study points to a dual focus: strengthening resources while helping drivers keep demands within a manageable range. First, building and sustaining support ecosystems through family encouragement, peer communities, and trusted social networks remains central because social support is repeatedly linked to better wellbeing outcomes (Soulsby and Bennett, 2015; Fisher and Cassady, 2019). Second, promoting work-life balance through stronger boundary-setting habits, recovery routines, and realistic scheduling may be particularly impactful because it supports drivers’ sense of control and helps prevent time pressure from escalating into ongoing strain (Sonnentag and Fritz, 2007). Finally, the findings caution against treating workload or time pressure as inherently harmful. In this context, pressure may still coexist with wellbeing when drivers experience autonomy, can recover adequately, and perceive their work as meaningful (Gehrke et al., 2019). Therefore, the practical priority is not only reducing demands, but also ensuring that drivers have the psychological and social resources needed so that high-demand periods remain “manageable challenges” rather than becoming persistent sources of distress (Bakker and Demerouti, 2007; Crawford et al., 2010).

## Theoritical contributions

The findings of this study offer several significant contributions to the theoretical landscape of organizational psychology and digital labor studies, particularly within the context of the emerging gig economy in Southeast Asia (Fotiadis et al., 2019). Extension of the JD-R Model in Non-Traditional Work Settings First, this research extends the Job Demands-Resources (JD-R) model by applying it to informal gig workers, a group often neglected in classical organizational theory. While Park and Searcy (2012) and Saragih et al. (2021) emphasize that job autonomy is a primary driver of wellbeing in formal settings, our study reveals a more nuanced autonomy-stress paradox (Das et al., 2015). This study is contribute to the theory that in the gig economy, autonomy does not always act as a simple resource; rather, it is often intertwined with workload and time pressure, creating an “ambivalent subjective experience” where the freedom to work is shadowed by the necessity of constant availability (Dillon and Wibowo, 2020).

This study identifies time pressure as a critical mediating mechanism between workload and psychological wellbeing. This adds theoretical depth to the observations made by Wang et al. (2022) regarding financial precarity. While previous research identified *that* gig workers are stressed, our findings explain *how* specifically through the compression of time dictated by platform algorithms. This contributes to “Algorithmic Management Theory” by showing that psychological strain is not merely a byproduct of physical labor, but a result of the temporal discipline imposed by digital interfaces, as suggested by Zhang et al. (2022).

The study bridges the gap between individual psychological resources and macro-level structural support. By integrating the perspectives of Radzi et al. (2022) and Rahim et al. (2021), we provide empirical evidence that “Social Support” serves as more than just an interpersonal resource; it is a vital buffer against the unique stressors of the digital workforce (Connolly et al., 2020). This finding suggests that for gig workers, wellbeing is not solely an individual achievement but is deeply rooted in the strength of their social and professional networks, reinforcing the need for “Community-Based Support” theories in the post-pandemic digital era (Esteban et al., 2022).

## Managerial implications

Based on evidence from 267 Gojek drivers in Jakarta, the managerial implications suggest that driver wellbeing can remain strong even under high workload and time pressure when drivers have sufficient resources to interpret, manage, and recover from daily demands. Therefore, Gojek’s priority should not only be reducing workload, but also designing a work experience that stays challenging, meaningful, and humane by strengthening key resources and improving system-level support.

First, because social support has a significant positive association with psychological wellbeing, Gojek should reinforce a structured community ecosystem that goes beyond communication channels and functions as a peer-support and learning space. This can be done through mentoring, facilitated group sessions, and community-based problem solving, which is especially relevant in platform work that can feel individual and algorithm-driven.

Second, since work-life balance is positively linked to wellbeing, Gojek can protect mental health and retention by helping drivers use flexibility effectively. Practical financial education can reduce the need for excessive working hours, while simple guidance on boundary setting and recovery routines can help drivers sustain healthier working patterns. This is consistent with evidence that better work-life balance reduces stress and improves life satisfaction (Rahim et al., 2023; Shagvaliyeva and Yazdanifard, 2014).

Third, because workload can function as a motivating “challenge demand” when supported by adequate resources, the managerial focus should shift toward fairness and clarity rather than simply lowering workload. More transparent and predictable order distribution and incentive rules can strengthen drivers’ perceptions that effort is met with proportionate rewards, which aligns with JD-R logic and prior findings on challenge demands (LePine et al., 2005; Zappalà et al., 2022).

Fourth, given that time pressure also shows a positive association with wellbeing when it remains controllable, Gojek should protect driver autonomy and add lightweight micro-training on time management and coping strategies. This can help drivers stay focused without tipping into chronic strain (LePine et al., 2005; Mondo et al., 2023).

Finally, because time pressure does not mediate the effect of social support on wellbeing, interpersonal support alone is unlikely to reduce urgency that is driven by algorithmic and system features. Gojek should therefore complement community initiatives with system-oriented solutions such as clearer in-app scheduling controls, rest prompts, and features that help drivers regulate working rhythms (Irawan et al., 2019). At the same time, since time pressure mediates the effects of workload and work-life balance on wellbeing, these interventions should be integrated as a coherent “resource bundle” that combines flexibility tools, stress-management education, and community support to sustain psychological wellbeing without undermining productivity.

## Conclusion

This study examined the effects of social support, work-life balance, and workload on psychological wellbeing, and tested time pressure as a mediating variable among Gojek drivers in Jakarta using Structural Equation Modeling (SEM). The findings show that social support has a positive and significant effect on psychological wellbeing, indicating that support from family, peers, and driver communities functions as a key job resource that strengthens psychological resilience and social connectedness. Work-life balance also positively and significantly predicts psychological wellbeing, suggesting that drivers who can manage work and personal life more proportionally experience lower stress and higher life satisfaction.

Interestingly, workload has a positive and significant relationship with psychological wellbeing in this sample, suggesting that high workload was not uniformly experienced as harmful in this context. Rather than indicating that workload is inherently beneficial, this result may reflect the possibility that some drivers appraise intensive work as meaningful, productive, or linked to daily achievement, particularly when they retain sufficient control and support. Regarding mediation, time pressure does not significantly mediate the relationship between social support and psychological wellbeing, which suggests that interpersonal support improves wellbeing without necessarily reducing time pressure that is largely shaped by algorithmic and systemic job conditions. However, time pressure significantly mediates the relationships between workload and psychological wellbeing and between work-life balance and psychological wellbeing, indicating that time pressure represents an important mechanism through which job demands and resources translate into drivers’ wellbeing outcomes. Finally, time pressure itself has a positive and significant effect on psychological wellbeing, showing that when it remains moderate and controllable, it can act as a motivating force that enhances focus, perceived competence, and satisfaction at work.

### Limitation and future directions

Based on the study’s findings and conclusions, several limitations and future research directions can be outlined to strengthen the academic contribution and extend the understanding of psychological wellbeing among gig workers, particularly platform-based drivers.

A first limitation is that this study used convenience sampling in a highly mobile informal-worker population, which may limit representativeness and introduce sampling bias. In addition, all variables were measured using self-report responses collected at a single point in time, meaning that common method bias cannot be fully ruled out and causal interpretation remains limited. Although social support, work-life balance, and workload were found to influence psychological wellbeing, this study did not examine the specific contribution of each social support dimension, namely family, friends, and significant others. Future studies are encouraged to conduct dimension-level analyses to identify which source of support most strongly predicts psychological wellbeing and under what conditions it becomes most salient. In addition, this study employed a cross-sectional design, which restricts causal inference and does not capture how psychological wellbeing may evolve over time. Longitudinal research is therefore recommended to observe drivers’ adaptation processes to work pressure, income changes, and shifting work-life boundaries in the longer term.

Future research can also expand the comparative context to increase the generalizability and theoretical richness of findings. Comparative studies across gig workers, formal employees, and hybrid workers would provide deeper insight into how different work structures, levels of organizational control, and income stability shape psychological wellbeing. Finally, this study did not incorporate broader structural and external factors, such as macroeconomic conditions, fuel price fluctuations, and platform policy changes, which may amplify or reduce drivers’ work pressure and time constraints. Incorporating these systemic variables in future models would help move the discussion beyond individual-level explanations and offer a more comprehensive account of how psychological wellbeing is shaped within the broader ecosystem of platform work.

## Data Availability

The raw data supporting the conclusions of this article will be made available by the authors, without undue reservation.
